# Update on the controversial identity of cells expressing *cnr2* gene in the nervous system

**DOI:** 10.1111/cns.13977

**Published:** 2023-01-05

**Authors:** Wanda Grabon, Jacques Bodennec, Sylvain Rheims, Amor Belmeguenai, Laurent Bezin

**Affiliations:** ^1^ Lyon Neuroscience Research Center TIGER Team Bron France; ^2^ Lyon 1 University CNRS UMR 5292, Inserm U1028 Villeurbanne France; ^3^ Epilepsy Institute IDEE Bron France

**Keywords:** astrocytes, CB2R tissue distribution, immunodetection, microglia, neurons

## Abstract

The function of cannabinoid receptor type 2 (CB2R), mainly expressed by leukocytes, has long been limited to its peripheral immunomodulatory role. However, the use of CB2R‐specific ligands and the availability of CB2R‐Knock Out mice revealed that it could play a functional role in the CNS not only under physiological but also under pathological conditions. A direct effect on the nervous system emerged when CB2R mRNA was detected in neural tissues. However, accurate mapping of CB2R protein expression in the nervous system is still lacking, partly because of the lack of specificity of antibodies available. This review examines the regions and cells of the nervous system where CB2R protein is most likely present by cross‐referencing mRNA and protein data published to date. Of the many antibodies developed to target CB2R, only a few have partially passed specificity tests and detected CB2R in the CNS. Efforts must be continued to support the development of more specific and better validated antibodies in each of the species in which CB2R protein is sought or needs to be quantified.

## Graphical abstract

Presence of the CB2R receptor in the nervous system: where do we stand? This review outlines the brain regions and cells where its expression is most likely.

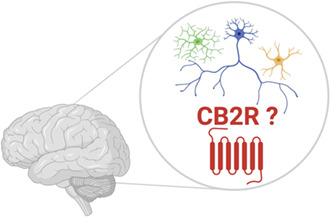

## 1 INTRODUCTION

The endocannabinoid system is recognized as an important player in neuromodulation in the central nervous system (CNS). It comprises cannabinoid receptors, endogenous molecules called endocannabinoids (eCBs) that activate these receptors, and enzymes that synthesize and degrade eCBs.^1^ The most abundant eCBs are anandamide and 2‐arachidoylglycerol. Many effects of eCBs are mediated by type 1 (CB1R) and type 2 (CB2R) cannabinoid receptors, which are the best known and involved in the homeostatic control of several physiological functions in the brain and other organs.^2^ CB1R and CB2R are G protein‐coupled receptors (GPCRs) that, in addition to interacting with eCBs, are also activated by synthetic and plant‐derived cannabinoids. Both were cloned in the early 1990s from human leukemia cells.^3^, ^4^ However, it is important to note here that we must take a much broader view of this system. Indeed, studies over the last decade have revealed the existence of a wide range of lipid mediators with eCB‐like properties, novel enzymes, and new receptors, effectively complicating our picture of the endocannabinoid system and justifying the use of endocannabinoidome to describe it.^5^

CB1R is the most prevalent GPCR in the CNS and is expressed extensively by most neuron types.^6^ This receptor is the major mediator of the psychoactive effects of *Cannabis sativa* and its derivatives. It has been cloned from rat, mouse, and human tissues and exhibits 97%–99% amino acid sequence identity across these species. The intracellular region of CB1R is most frequently coupled to G_i/o_ proteins. Stimulation of CB1R by endogenous or exogenous agonists inhibits adenylate cyclase (AC) activity with subsequent reduction in intracellular levels of cyclic adenosine monophosphate or promotes mitogen‐activated protein kinase (MAPK) activity. The different intracellular pathways involved have been previously described in greater detail.^7^

CB2R exhibits 44% homology with CB1R.^8^ Unlike CB1R, CB2R is mainly expressed by peripheral immune cells and mediates immunomodulatory properties. Due to this near‐exclusive expression, CB2R has long been referred to as a peripheral cannabinoid receptor.^9^ However, since the early 2000s, an increasing number of studies have reported the effects of CB2R‐specific ligands on neuroinflammation. In addition, such ligands have also been shown to exert effects at the neuronal and behavioral levels both in physiological and pathological conditions,^10^, ^11^, ^12^ suggesting that CB2R is also expressed in the CNS and may represent a therapeutic target for many pathologies of the nervous system. Owing to technical advances and the emergence of more sensitive methods, weak *CB2R* gene expression has been confirmed in the brain, paving the way for attempts to refine CB2R messenger ribonucleic acid (mRNA) and protein detection at both tissue and cellular levels. However, numerous controversies regarding the irrelevance of the detection tools used have led to the current “CB2R identity crisis.”^13^ Resultantly, no consensus exists regarding the mapping of CB2R expression in the nervous system.

In this review, we attempted to understand what underlies these difficulties and list the brain areas and cells where *CB2R* gene expression is most likely, by cross‐referencing transcript and protein detection data.

## 2 CB2R: FROM GENE TO PROTEIN

The *CB2R* gene and receptor structures have been recently detailed by Jordan et al.^10^ Briefly, the human *cnr2* gene size reaches 90 kb, whereas the size of the mouse and rat *cnr2* gene is 23 and 20 kb, respectively. Mouse and rat CB2R proteins share 93% amino acid homology, and human CB2R shares 82% homology with mouse and 81% homology with rat.^14^ Some characteristics of the human, mouse, and rat CB2R transcripts and proteins are summarized in Table S1. Given that the identification of the different isoforms of CB2R transcript and protein has evolved several times since the discovery of the gene and the protein and has not been taken into consideration in the vast majority of the studies, we chose not to distinguish them in this review.

CB2R is a membrane protein expressed at the plasma membrane. Recently, it has been demonstrated that CB2R is localized in the nonlipid raft compartment of the plasma membrane of mouse cortical tissue.^15^ CB2R also mediates its effects in an internalized form. Intracellular injections of specific agonists elicited Ca^2+^ signaling,^16^ which contributes to the very complex pharmacology of this receptor. Similar to CB1R, CB2R is a GPCR that is mainly coupled to G_i/o_ α proteins. Its stimulation inhibits AC activity and activates MAPK.^17^ CB2R intracellular pathways have been recently reviewed.^2^ Intracellular signaling pathways may differ depending on the tissues, cell types,^18^ and subcellular localization of CB2R.

## 3 DETECTION OF CB2R IN THE CNS: METHODOLOGICAL CONSIDERATIONS

### 3.1 At the mRNA level

*Cnr2* gene expression detection in the CNS was first attempted at the transcriptional level during the 1990s using endpoint reverse transcription polymerase chain reaction (RT‐PCR) and northern blots. Numerous studies have failed to detect CB2R mRNA in the CNS of human,^19^, ^20^ mouse,^21^, ^22^ and rat.^4^, ^21^, ^23^, ^24^ It is noteworthy that the use of endpoint RT‐PCR presents many pitfalls in detecting and quantifying mRNAs in biological samples.^25^ Since then, real‐time or quantitative PCR (qPCR) has emerged as a robust and widely used methodology for biological investigation and has resulted in greater accuracy in the detection and quantification of CB2R‐mRNA in the CNS. Moreover, in situ hybridization (ISH)—in particular, the RNAscope *ISH* technique—which affords very high sensitivity and specificity^26^ in detecting in situ mRNA molecules—even expressed at very low levels—allows for the refinement of cartography of *Cnr2* gene expression in the CNS. In this review, only the results of studies that investigated CB2R‐mRNA expression using RT‐qPCR and/or ISH are presented.

### 3.2 At the protein level

Many antibodies have been developed to identify the protein expression and localization of CB2R. To be considered specific, an antibody should meet different criteria, including the absence of labeling in genetic knockout (KO) animals, validation by western blotting (WB), and the ability to be blocked by the immunizing peptide used to generate the antibody. Ideally, concordant results should be obtained with antibodies raised against other epitopes when available. The “CB2R identity crisis” ^13^ partly relies on the lack of fully validated and commercially available antibodies. Thus, for a given species, the absence of a signal should be obtained in KO animals with complete invalidation of the *Cnr2* gene established in the same species. However, only two mouse strains presenting with partial deletion of the *Cnr2* gene encoding either the C‐terminal (Zimmer strain) or N‐terminal (Deltagen strain) of the protein (partial CB2R‐KO mice) have been generated to date. To test the specificity of any anti‐CB2R antibody, it has recently been advised to carefully select the partial CB2R‐KO mouse strain with a deletion of the gene sequence encoding the 3D structure of the epitope recognized by the antibody to be tested.^27^

Since the first rabbit polyclonal antibody raised against a C‐terminal peptide sequence of human CB2R in 1995 by Galiègue et al.,^20^ different polyclonal antibodies have been generated to detect CB2R in the primate and rodent nervous tissue. In this review, which is not exhaustive, we identified 22 different anti‐CB2R antibodies used in immunohistological studies targeting the CNS. For ease of reading, an upper‐ or lower‐case letter has been assigned to each antibody depending on whether the authors mentioned the precise reference or not, respectively. (Table 1).

**TABLE 1:** List of anti‐CB2R antibodies used for the detection of CB2R protein in the central nervous system

Ab	Ref.	Distri.	Availability	Immunogen	Host	Poly‐mono clonal	Predicted Species reactivity
A	#101550	Cayman Chemical	Yes	aa 20–33 of human CB2R	Rabbit	Poly	Human, mouse
B	#Ab3561	Abcam	Yes	aa 1–32 of rat CB2RR	Rabbit	Poly	Human, rat
C	#Ab45942	Abcam	No	aa 200–300 of rat CB2R	Rabbit	Poly	Human, mouse, rat
D	#ACR‐002	Alomone	Yes	aa 228–242 of rat CB2R	Rabbit	Poly	Mouse, rat
E	#ACR‐003	Alomone	No	aa 11–24 of human CB2R	Rabbit	Poly	Human
F	#bs‐2377R	Bioss	Yes	aa 298–360 of human CB2RR	Rabbit	Poly	Human, mouse, rat
G	#CB2R2A	Alpha diagnotics	Yes	C‐term of rat CB2R	Rabbit	Poly	Rat
H	#KMCB2R‐CT	Ken Mackie	No	aa 326–342 of rat CB2R	Rabbit	Poly	Rat
I	#KMCB2R‐NT	Ken Mackie	No	N‐term of rat CB2R	Rabbit	Poly	Rat
J	#MAB36551	R&D Systems	No	aa 1–360 of human CB2R	Mouse	Mono	Human
K	#NIH‐5633	Customed (Genemed Synthesis)	No	C‐term of mouse CB2R	Rabbit	Poly	Mouse
L	#PA1‐744	Affinity Bioreagents	No	aa 1–33 of human CB2RR	Rabbit	Poly	Human
M	#PA1‐746	Affinity Bioreagents	Yes	aa 1–32 of rat CB2R	Rabbit	Poly	Human, rat
N	#SAB2500191	Sigma‐Aldrich	Yes	C‐term of human CB2R	Goat	Poly	Human
O	#sc10071	Santa Cruz Biotechnology	No	N‐term of human CB2RR	Goat	Poly	Human
P	#sc10073	Santa Cruz Biotechnology	No	C‐term of human CB2R	Goat	Poly	Human
Q	#sc10076	Santa Cruz Biotechnology	No	C‐term of mouse CB2RR	Goat	Poly	Mouse, rat
R	#sc25494	Santa Cruz Biotechnology	No	aa 301–360 of human CB2R	Rabbit	Poly	Human, mouse, rat
s	?	Affinity Bioreagents	—	N‐term peptide	Rabbit	Poly	—
t	?	Affinity Bioreagents	—	—	—	Poly	—
u	?	Sigma‐Aldrich	—	—	Rabbit	Poly	—
v	?	—	—	N‐term peptide	‐	Poly	—

*Note*: For the ease of reading, an upper or a lower‐case letter has been assigned to each antibody depending on whether authors mentioned the precise reference or not, respectively. The availability of the antibodies described on the day the review was written is specified in the “availability” column.

Abbreviations: AA, amino‐acid; Ab, antibody; CB2R, cannabinoid receptor type 2; Distrib, distributor; Ref, commercial reference.

#### 3.2.1 Antibodies for which part of the validation was performed in partial‐KO mice

**FIGURE 1:**
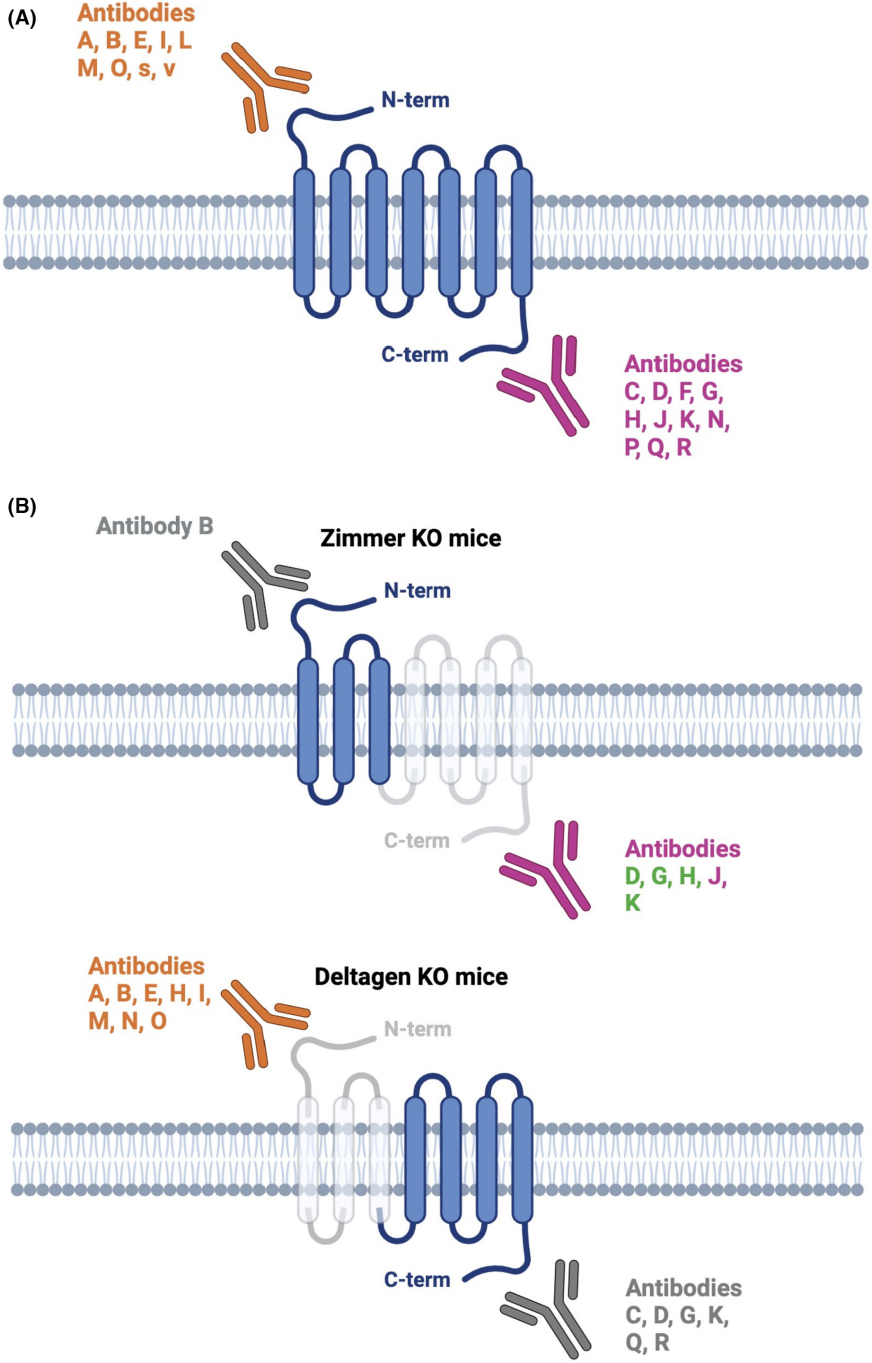
Anti‐CB2R antibodies. The letters assigned to the antibodies correspond to those assigned in Table 1. (A) Recognition of CB2R by antibodies used on nervous tissue. Antibodies directed against the N‐terminal (N‐term) region of the CB2R receptor are shown in orange. Antibodies against the C‐terminal (C‐term) region of the receptor are shown in pink. (B) CB2R antibodies tested in CB2R partial Knock‐Out mice. The antibodies which have been tested on tissue from each of the two available partial KO mouse are represented, that is on the Zimmer strain^28^ in which CB2R C‐term region is deleted and on the Deltagen strain (The Jackson Laboratory, *Cnr2* ^*tm1Dgen*^/J, #005786) in which CB2R N‐term region is deleted. The antibodies tested on tissue from “non‐adequate” partial KO mice, that is to say in which the deleted part of the CB2R gene does not encode the epitope recognized by the antibody, are represented in gray. Conversely, the antibodies which have been tested on “adequate” partial KO mouse tissue are represented in colors, that is, in orange and pink for those recognizing the N‐term and C‐term regions, respectively. The antibodies validated by these tests, that is to say for which a reduction in signal was observed on the tissue of the appropriate KO mouse, are represented in green. C‐term, C‐terminal; CB2R, cannabinoid receptor type 2; KO, knock‐out; N‐term, N‐terminal. Figure generated with https://biorender.com/

The best negative control for testing the specificity of any anti‐CB2R antibody in mice are full‐length CB2R‐KO mice. However, these mice are currently unavailable to date. Instead, two strains of mice with partial deletions of the *CB2R* gene have been generated. One has a deletion of the sequence encoding the N‐terminal part of the protein and is often referred to as the Deltagen strain (The Jackson Laboratory, *Cnr2* ^*tm1Dgen*^/J, #005786). In contrast, the other has a deletion of the sequence encoding the C‐terminal part of the protein, commonly referred to as the Zimmer strain.^28^ The two mouse strains were used to determine the specificity of the signals obtained using antibodies generated against the CB2R protein. Nevertheless, it has been hypothesized that mutant or truncated fragments of CB2R are present in mice with a partial deletion of the CB2R gene, making interpretation of the results difficult, particularly when the antibodies were tested in mice whose *CB2R* gene deletion does not match the portion of the protein recognized by these antibodies.^27^ In the following section, we will only describe the validation tests performed in partial‐KO mice whose deleted sequence encodes the epitope recognized by the tested antibody. The antibodies have been represented according to their recognition of the N‐terminal or C‐terminal region of CB2R in Figure 1A. The use of different antibodies in partial‐KO mice is illustrated in Figure 1B.

*D*, *G*, *H*, and *K* antibodies have been tested in the suitable Zimmer mouse strain. The use of this strain has revealed reduced in situ immunolabeling signal (*D*, *K* ^27^, ^29^; *G* ^30^, ^31^). WB and/or the loss of in situ immunolabeling with the immunizing peptide support the specificity of *D*,^27^, ^29^ *H*,^27^ and *K* ^27^, ^29^ antibodies to detect CB2R in mice (Table 2). For antibodies *A*, *B*, and *M*, tested in the suitable Deltagen mouse strain, less intense in situ immunolabeling was observed (*A and M* ^32^; *B* ^27^, ^33^); however, such a validation has not been identified by others (A^34^; B^34^, ^35^). Additionally, WB analyses revealed that the three antibodies failed to detect a reduced signal in the Deltagen mouse strain (*A and M* ^32^, ^34^; *B* ^27^). Notably, the sensitivity and specificity of *A*, which appears to be one of the most widely used antibody for CB2R detection in the CNS, have been extensively studied by Marchalant et al. using combined WB and mass spectrometry. They unequivocally demonstrated its sensitivity in overexpressing cell lines, but also showed cross‐reactivity with other proteins, resulting in a lack of specificity.^36^

**TABLE 2:** Evaluation of the validity of antibodies used for the detection of CB2R in the central nervous system

AB	Predicted species reactivity	Validation tool				Applications		Studies	
		BP	WB	KO mice		IH	WB	Model	References
				Adequate	Inadequate				
A	Human, mouse					x		Human	[88]
		−				x		Human fœtus	[56]
		−	+			x		NHP	[60]
		−	+	+			x	Mouse	[32]
		−		−		x			
			−	+		x	x	Mouse	[34]
						x		Mouse	[89]
			+				x	Rat	[42]
		−	+			x		Rat	[41]
			+				x	Rat	[40]
						x		Mouse	[90]
						x		Human	[76]
B	Human, (mouse,) rat				+	x		Mouse	[29]
			−	+		x	x	Mouse	[34]
					−	x		Mouse	[31]
		−	+	−	+		x	Mouse	[27]
				−	+	x			
				−		x		Mouse	[33]
		−	+	+		x		Rat	[35]
						x		Rat	[42]
			+			x		Rat	[44]
			+			x		Rat	[91]
								Mouse	[74]
		−	+				x	Rat	[43]
						x		Rat	[92]
						x		Rat	[93]
C	Human, mouse, rat		+		+		x	Mouse	[34]
		−				x		Mouse	[37]
		−				x		Rat	[37]
		−				x		Rat	[47]
		−				x		Rat	[94]
D	Mouse, rat	−		−		x		Mouse	[29]
		−	+	−	+		x	Mouse	[27]
				−	+	x			
						x		Mouse	[33]
			+				x	Rat	[81]
E	Human		−	+			x	Mouse	[34]
F	Human, mouse, rat	−				x		Mouse	[39]
G	Rat	+	−			x		Mouse	[32]
			+	+			x	Mouse	[34]
		−	+				x	Rat	[30]
						x			
		−	+				x	Ferret	
						x			
				−		x		Mouse	
				−		x		Mouse	[31]
H	Rat		−		+		x	Mouse	[32]
		−			+	x			
			+	−			x	Mouse	[27]
		−				x		Rat	[48]
						x		Rat	[82]
						x		Rat	[80]
I	Rat		−	+			x	Mouse	[32]
		+		+		x			
J	Human	−				x		Human	[57]
		−				x		Human	[95]
K	Mouse	−		−		x		Mouse	[29]
		−	+	−	+		x	Mouse	[27]
				−	+	x			
L	Human					x		NHP	[96]
			+			x		Human	[52]
		−	+			x		Human	[50]
						x		Human	[97]
		−				x		Human	[53]
			+			x	x	Human	[51]
M	Human, rat	−	−	+			x	Mouse	[32]
		+		−		x			
			+	+			x	Mouse	[34]
						x		Rat	[98]
		−				x		Rat	[49]
						x		Mouse	[44]
N	Human		−		+		x	Mouse	[32]
					+	x			
O	Human	−	+	+		x		Rat	[35]
		−	+			x		Rat	[45]
P	Human					x		Human	[59]
		−	+			x		Rat	[45]
			+				x	Human	[54]
Q	Mouse, rat				+	x		Mouse	[34]
			+		+		x		
		−	+			x		Rat	[45]
		−	+			x		Mouse	[38]
						x		Mouse	[57]
						x		Rat	[99]
R	Human, mouse, rat		+		+		x	Mouse	[34]
						x		Human	[59]
s			+			x	x	Human	[55]
t		−				x		Human	[58]
u		−		−		x		Rat	[41]
v						x		Mouse	[66]

*Note*: Nonexhaustive list of studies that have used antibodies to detect CB2R in the central nervous system in situ (by immunohistochemistry) or on homogenates (by western blot) and that have tested or not their specificity through the use of available partial KO mice (Deltagen strain and Zimmer strain) and/or the use of blocking peptide and/or western blot. The letters assigned to the antibodies correspond to those assigned in Table 1. For each validity test, the symbol “−” stands for an absence or a decrease in the measured signal. Thus, for validity tests with blocking peptide, symbol “−” indicates a decrease in signal on histological sections (for IH studies) or on homogenates (for WB studies) in the presence of the peptide. Similarly, for tests on partial KO mice, the symbol “−” indicates a decrease of the signal on histological sections (for IH studies) or on homogenates (for WB studies) from KO mice compared to that measured in wild type animals. Finally, for western blot studies, the symbol “−” indicates the absence of a band at the size corresponding to CB2R. Conversely, the “+” symbol corresponds to the presence of a signal. For validity tests with blocking peptide or on KO mice, it indicates a quantity of signal identical to that measured without peptide or on wild‐type mice, respectively. For western blot, it indicates the presence of a band corresponding to the molecular weight of CB2R. The validation tests conducted on KO mice that we have qualified as “adequate” correspond to those conducted on KO mice whose deleted sequence encodes the epitope recognized by the antibody tested.

For the validation tools, the results in favor of the specificity of the tested antibody for CB2R are represented in green (i.e. an absence of signal either with the blocking peptide or when using the adequate KO mouse, or the presence of a band in WB). The results invalidating the specificity of the antibody for CB2R are represented in red (presence of signal either with the blocking peptide or when using the adequate KO mouse, and absence of band in WB). Results that do not allow to conclude are shown in grey (use of inadequate KO mice).

Abbreviations: Ab, antibody (see Table 1); BP, blocking peptide; IH, Immunohistology; KO, knock out; NHP, nonhuman primate; WB, western blot.

Other antibodies have been invalidated when correctly tested in adequate partial‐KO mouse strains: *E*,^34^ *I*,^32^ and *O*.^35^

#### 3.2.2 Other antibody validation studies

Antibodies other than A, B, D, E, G, H, I, K, M, and O have not been tested in a suitable partial‐KO mouse strain. However, *C* and *Q* antibodies were validated in mice by WB and using a blocking peptide (*C* ^34^, ^37^; *Q* ^34^, ^38^). *C* antibody sensitivity was further confirmed in a knock‐in mouse model and in overexpressing cells.^37^ *R and F* antibodies were validated only by WB^34^ and blocking peptide,^39^ respectively. Finally, *N* antibody has not been validated by WB.^32^

In rats, *A*, *B*, *G*, *O*, *P*, and *Q* were validated by both WB and immunizing peptide (*A* ^40^, ^41^, ^42^; *B* ^35^, ^43^, ^44^; *G* ^30^; *O* ^35^, ^45^; *P* ^46^; *Q* ^45^) and *C*, *H*, *M*, and *u* were validated only by the use of their blocking peptide (*C* ^37^, ^47^; *H* ^48^; *M* ^49^; *u* ^41^).

In humans, *L* appears to be the most commonly used antibody to detect CB2R in the CNS. It has been validated using both WB and blocking peptide.^50^, ^51^, ^52^, ^53^ The other antibodies were only validated either by WB for *P* ^54^ and *S* ^55^ or by the use of a blocking peptide for *A*,^56^ *J*,^57^ and *t*.^58^ *R* was also used to detect CB2R protein in the human CNS, but its validity was not assessed.^59^ Finally, the *A* antibody was validated by WB in nonhuman primates (NHP).^60^

All presented antibodies are polyclonal, except for antibody *J*, which is a mouse monoclonal antibody. Considering the diversity of the antibodies presented and the obvious lack of consistency in the data obtained so far in the studies aimed at testing their specificity, it is now necessary to have anti‐CB2R monoclonal antibodies for each species detailed, at least tested on cells of the targeted species, with a complete KO of the *CB2R* gene.

The level of confidence attributed to the results of immunohistological studies depends strongly on the tools used to validate the antibodies used. Thus, we classified brain regions according to the degree of probability that they would express CB2R protein. The brain regions with the highest probability of expressing CB2R protein were those for which CB2R was detected with at least one antibody validated in the relevant partial‐KO mouse model, that is, antibodies D, G, H, and K. This is followed by brain structures for which the probability was lower because the antibody used has only been validated by WB or using a blocking peptide. Finally, we remained cautious about the possibility that CB2R could have been detected in certain brain regions, especially with antibodies whose specificity had not been demonstrated, while CB2R mRNA was not sought or detected, especially by ISH techniques. It remains to be mentioned that the absence of CB2R‐mRNA detection by RT‐qPCR in a homogenate of a brain area can be insufficient to reject the possibility that the CB2R protein is present in that brain region, because CB2R‐mRNA might be too diluted in the sample to reach the detection threshold.

## 4 BRAIN REGIONS AND CELL TYPES FOUND TO EXPRESS CB2R AT THE MRNA LEVEL

Under physiological conditions, CB2R mRNA has been detected using RT‐qPCR and/or ISH, including RNAscope technology, in the neocortex,^61^, ^62^, ^63^ nucleus accumbens,^14^, ^63^, ^64^ striatum,^14^, ^63^, ^64^, ^65^, ^66^ hippocampus,^34^, ^40^, ^63^, ^65^, ^66^, ^67^ amygdala,^61^, ^63^ Ventral Tegmental Area (VTA),^29^, ^68^, ^69^ red nucleus,^33^ substantia nigra,^70^ cerebellum,^41^, ^62^, ^66^ brainstem,^30^ spinal cord,^71^ hypothalamus,^41^, ^72^ and retina.^42^, ^73^

CB2R mRNA has also been detected in pathophysiological conditions in the neocortex in a model of stroke,^74^ in the striatum in models of Huntington's disease^49^ and Parkinson's disease (PD),^57^, ^75^ in the substantia nigra in a model of PD,^76^ and in the spinal cord in models of neuropathic chronic pain^77^ and multiple sclerosis.^78^ The detection of CB2R mRNA using quantitative/semi‐quantitative methods in these regions makes it very likely that the CB2R protein is also expressed.

To identify cells that express CB2R, some studies have performed double detection at the mRNA level and found that CB2R mRNA was expressed in tyrosine hydroxylase (TH)‐positive neurons of the VTA,^29^, ^64^, ^68^ glutamatergic neurons of the red nucleus,^33^ and CA3 neuronal nuclei (NeuN)‐positive neurons.^67^ Using combined ISH and immunohistochemistry, CB2R mRNA was also detected in pyramidal neurogranin‐positive neurons and glutamate decarboxylase (GAD)67‐positive gamma‐aminobutyric acid (GABA)ergic interneurons of the hippocampus^34^ and interneurons of the striatum,^65^ as well as in activated cluster of differentiation (CD)11b‐positive microglia of the spinal cord.^77^ CB2R mRNA has been detected in interneuron‐like cells in the hippocampus.^65^ CB2R mRNA was also detected in the microglial cells of the neocortex following fluorescent‐activated cell sorting.^79^ Finally, based on cell morphology, CB2R mRNA was also localized in neuron‐like cells in the substantia nigra.^70^

## 5 IDENTIFICATION OF BRAIN REGIONS WHERE BOTH CB2R MRNA AND PROTEIN WERE DETECTED

To express the CB2R protein, the brain regions and cell populations involved must express CB2R mRNA. In the previous section, we detailed the brain regions in which CB2R mRNA was unequivocally identified. In this section, we report studies that have demonstrated the presence of CB2R protein in these same regions, which will be classified according to whether the CB2R protein was detected with validated antibodies or with nonvalidated antibodies.

### 5.1 Brain regions and cell types with CB2R detection using validated antibodies

In this section, we provide a list of brain regions in which CB2R protein was detected by at least one of the antibodies that we currently consider the most specific, that is, D, G, H, and K antibodies.

#### 5.1.1 Hippocampus

CB2R expression has been extensively investigated in both the human and rodent hippocampi in physiological as well as pathological states.

##### Neurons

Under physiological conditions, CB2R protein has been detected in neuron‐like cells of the hippocampus using G and H antibodies. Based on the morphological features observed by electron microscopy, CB2R was detected using antibody H in CA1 neurons in rats.^48^ Using the same antibody, CB2R was detected in rats in both the dentate gyrus and stratum radiatum.^80^

Finally, CB2R neuronal expression has also been studied in cultured hippocampal neurons from both rats and mice using the validated D antibody.^81^

##### Microglia

Under physiological conditions, CB2R has been detected in rat microglia‐like cells, using antibody H.^48^

#### 5.1.2 VTA

Using antibodies D and K, CB2R protein has been detected in the VTA under physiological conditions in mouse neurons and astrocytes.^27^, ^29^

#### 5.1.3 Red nucleus

CB2R protein has been detected in TH‐positive neurons in the red nucleus, using antibody D.^33^

#### 5.1.4 Brainstem

In the mouse brainstem, under physiological conditions, NeuN‐positive cells co‐express CB2R in the dorsal motor nucleus of the vagus, nucleus ambiguous, and spinal trigeminal nucleus, using antibody G.^30^

#### 5.1.5 Retina

Immunolabeling of CB2R has been detected in different neuronal elements of the mouse retina, namely, in the outer nuclear, outer plexiform, and inner plexiform layers, using the validated G antibody.^32^ CB2R was also detected in the inner nuclear layer of rats using H.^82^

### 5.2 Brain regions and cell types where CB2R was detected using unvalidated antibodies

It is noteworthy that some of the antibodies whose specificity is disputable, owing to the lack of validation in adequate KO mice, have also been used successfully in the regions presented in Section 5.1 (see the first part of Table S2).

In some brain regions described in Section 4, where CB2R mRNA has been detected, CB2R protein detection has been investigated using unvalidated antibodies only, that is, A, B, C, E, F, I, J, L, M, N O, P, Q, R, s, t, u, and v. This is the case for the following areas, as detailed in the second part of Table S2: neocortex, nucleus accumbens, striatum, amygdala, substantia nigra, cerebellum, spinal cord, and hypothalamus.

## 6 BRAIN REGIONS AND CELL TYPES WITH POOR EVIDENCE OF CB2R PROTEIN PRESENCE

In this section, we discuss areas where the presence of CB2R mRNA has not been investigated, to our knowledge, and where CB2R protein has only been detected with unvalidated antibodies. These regions are the lateral habenula, corpus callosum, midbrain, pons nuclei, and cochlea. The CB2R detection in these areas is presented in Table S3.

## 7 CONCLUDING REMARKS

Given the role of CB2R in the functioning of the nervous system, numerous studies have attempted to map the brain regions and identify the cells where it is expressed. The evolution of detection and assay techniques for CB2R mRNA has confirmed its low expression under physiological conditions. At the protein level, difficulties were more numerous owing to the lack of specificity of the different antibodies produced. Of the 22 antibodies used for CB2R protein detection in the nervous system and listed in this review, only four (D, G, H, and K) were considered more specific than the others on the basis of the reduction of the signal in mice whose genetic sequence coding for the epitope recognized by these antibodies was invalidated. On the basis of tables in which we have tried to provide as much relevant information as possible, we leave it to the discretion of reader to appreciate the choice of antibodies used in certain studies and the choices to be made for conducting their own studies. However, we have provided a table (Table S4) summarizing the regions and cell types likely to express CB2R, based on the relevance of the validation performed to test the specificity of the antibodies used, and on the detection of mRNA in these regions and cell types as detailed in Section 4.

Due to the difficulty in determining the localization and phenotype of CB2R‐expressing cells in the CNS using anti‐CB2R antibodies, Schmöle et al. generated a reporter mouse line in which the cnr2 gene is replaced by enhanced green fluorescent protein (EGFP) without interfering with its putative promoter sequences.^83^ Western blot assays on brain tissue samples showed no expression of GFP protein, but in situ GFP immunolabeling was present in some Iba‐1 expressing microglial cells in the hippocampus.

Recently, Lopez et al. generated a new transgenic mouse model with EGFP reporter gene expression under the control of the endogenous mouse *cnr2* gene promoter, referred to as CB2^EGFP/f/f^ mice. This approach allows for coupling of EGFP expression to cnr2 gene transcription without loss or modification of the CB2 protein. Microscopic analysis in adult CB2^EGFP/f/f^ mice has revealed no EGFP immunoreactivity in the brain^84^ and spinal cord.^84^, ^85^ However, when these mice were crossed with 5xFAD mice modeling Alzheimer's disease, an intense EGFP signal, and thus likely CB2R, was visualized in the offspring in the vicinity of beta‐amyloid plaques in cells with an ameboid shape reminiscent of activated microglia. Further investigations by electron microscopy revealed the presence of low EGFP signal in the membrane of some Iba1‐positive cells in the subiculum of CB2^EGFP/f/f^ mice, and at higher levels in both membrane and cytosol of Iba1‐positive cells from CB2^EGFP/f/f^/5xFAD mice.^86^ In retina, EGFP signal was sparse at baseline in microglial elements, but upregulated following inflammatory stimuli.^87^

Efforts must be made to support the development of better‐validated antibodies in each species in which the CB2R protein is assessed or must be quantified. However, while waiting for these tools of major importance, progress can be made toward refining the identification of cells expressing *CB2R*, at least at the transcript level, via assays performed on single cells or on specific cell populations obtained after the dissociation of different brain regions.

## CONFLICT OF INTEREST

The authors declare no conflict of interest.

## Supporting information


Supinfo

## Data Availability

Data sharing is not applicable to this article as no new data were created or analyzed in this study.
