# Supplementary material for: Update on the controversial identity of cells expressing cnr2 gene in the nervous system
Source: CNS Neurosci Ther. 2023 Jan 5;29(3):760–70. doi: 10.1111/cns.13977 (PMC9928557; doi:10.1111/cns.13977)
Supplement: Supplementary file 1 — Supinfo [file CNS-29-760-s001.docx]

**Update on the controversial identity of cells expressing *cnr2* gene in the nervous system**

**Supplementary Material**

**Running title:** CB2 receptor in the nervous system

**Supplementary Table 1. CB2R transcripts and proteins in human, mouse and rat.**

| **SPECIES** | **CHROMO-SOME** | **NC** | **TRANSCRIPT** | | | | | | | **PROTEIN** | | |
| --- | --- | --- | --- | --- | --- | --- | --- | --- | --- | --- | --- | --- |
|  |  |  | **NM** | **TRANSCRIPT VARIANT** | **NT** | **EXON 1** | **EXON 2** | **EXON 3** | **CDS** | **NP** | **AA** | **ISOFORM** |
| **Human** | 1 | NC_000001.11 | NM_001841.3 |  | 5265 | 1-117 | 118-5265 |  | 163-1245 | NP_001832 | 360 |  |
| **Mouse** | 4 | NC_000070.7 | NM_009924.4 | Variant 1 | 4084 | 1-431 | 432-4084 |  | 478-1521 | NP_034054.3 | 347 |  |
|  |  |  | NM_001305278.1 | Variant 2 | 3906 | 1-253 | 254-3906 |  | 300-1343 | NP_001292207.1 |  |  |
| **Rat** | 5 | NC_051340.1 | NM_001164143.3 | Variant 1 | 3300 | 1-220 | 221-3330 |  | 271-1353 | NP_001157615.1 | 360 | 1 |
|  |  |  | NM_001164142.3 | Variant 2 | 3198 | 1-118 | 119-3198 |  | 169-1251 | NP_001157614.1 |  |  |
|  |  |  | NM_020543.4 | Variant 3 | 1728 | 1-1029 | 1030-1114 | 1115-1728 | 1-1233 | NP_065418.3 | 410 | 2 |

Abbreviations: AA, amino-acids; CDS, coding sequence; NT, nucleotides.

**Supplementary Table 2: Brain regions where both CB2R mRNA and protein were detected, using unvalidated antibodies.**

| **REGIONS** | | **NEURONS** | **MICROGLIA** | **ASTROCYTES** | **OTHER CELLS** |
| --- | --- | --- | --- | --- | --- |
|  |  |  |  |  |  |
| **1. Regions where CB2R was also detected with validated antibodies** | **HIPPOCAMPUS** | **PHYSIOLOGICAL** : Neuron Specific Enolase-positive pyramidal neurons in CA2 and CA3, and, to a lesser extent, in the subiculum in rats^41^ with Antibodies A and u. NeuN-positive neurons in CA3^35,44,91^, CA1 and DG^44,91^with antibody B. Neuronal like-cells in CA2^41,85^ with antibody M. Cultured mouse hippocampal cells with antibody v^66^. **PATHOLOGICAL** : rat model of TLE, NeuN-positive cells in CA1, CA3 and DG 1h to 7 days following lithium pilocarpine-induced status epilepticus^44,91,93^ with antibody B. | **PHYSIOLOGICAL** : Rat parenchymal^44^ Iba1-positive cells with antibody B. Mouse perivascular Iba1-positive cells^38^ with antibody Q. **PATHOLOGICAL** : Rat model of TLE, Iba1 positive cells^44^ with antibody B. Stroke rat model, CD45-positive cells^45^ with antibodies O and Q. Mouse model of AD, Iba1-positive cells^40^ with antibody M. |  |  |
|  | **VTA** | **PHYSIOLOGICAL** : TH-positive neurons^29,90^ with antibodies A and B |  | **PHYSIOLOGICAL** : Astrocytes^90^ with antibodies A and B. |  |
|  | **RED NUCLEUS** | **PHYSIOLOGICAL** : Magnocellular neurons with antibody B^33^. |  |  |  |
|  | **RETINA** | **PHYSIOLOGICAL** : Inner segment of photoreceptors, in the inner nuclear layer and in the ganglion cell in rats^42^ with antibody B. Glutamine synthetase-positive cells i.e. Muller cells in NHP^60^ with antibody A. |  |  |  |
| **2. Regions where CB2R has been detected by non-validated antibodies only** | **NEOCORTEX** | **PHYSIOLOGICAL** : Rat pyramidal-like cells of the orbital cortex, the visual cortex, the motor cortex, and the auditory cortex^41^ with antibodies A and u. | **PHYSIOLOGICAL** : Mouse perivascular Iba1-positive cells^38^ with antibody Q. **PATHOLOGICAL** : Mouse model of stroke, activated microglial cells^74^; MDMA-induced neuroinflammation in rats, activated microglial cells^92^ with antibody B. Virus-Induced Encephalitis in NHP, microglial cells^96^, AD patients^50^ Down Syndrome patients^53^ with antibody L. AD patients,^58^ with antibody t. Rat stroke model, CD-45 positive cells^45^ with antibodies O and Q. | **PATHOLOGICAL** : GFAP-positive cells in the cortex of human fetus presenting focal cortical dysplasia^56^ with antibody A. | **PATHOLOGICAL** : Mouse stroke model, neutrophils^74^ with antibody B. Mouse model of traumatic brain injury, CD11b^+^CD45^high^ infiltrating macrophages^39^ with antibody F. |
|  | **NUCLEUS ACCUMBENS** | **PHYSIOLOGICAL** : NeuN-positive cells^90^ with antibody A. |  | **PHYSIOLOGICAL** : GFAP-positive cells^90^ with antibody A. |  |
|  | **STRIATUM** | **PHYSIOLOGICAL** : Neurons^66^ with antibody v. | **PATHOLOGICAL** : PD mouse model, Iba1-positive cells^57^ with antibody Q. PD patients, Iba1-positive cells^57^ with antibody J. Rat model of HD: CD11b-positive cells^49^ with antibody M. |  | **PATHOLOGICAL** : HD patients, endothelial-like cells^88^ with antibody A. |
|  | **AMYGDALA** | **PHYSIOLOGICAL** : Rat basolateral and basomedial amygdala^99^ with antibody Q. |  |  |  |
|  | **SUBSTANTIA NIGRA** |  | **PATHOLOGICAL** : PD patients, CD11b-positive cells^76^ with antibody A. | **PATHOLOGICAL** : PD patients, GFAP-positive cells^76^ with antibody A. PD patients, astroglial-like cells^57^ with antibody J. |  |
|  | **CEREBELLUM** | **PHYSIOLOGICAL** : Rat NSE-positive cells^41^ with antibody A and u. Rat neuronal-like cells of the granule layer^45^ with antibody P. | **PHYSIOLOGICAL** : Human perivascular microglial cells^52^ with antibody L. |  |  |
|  | **SPINAL CORD** | **PATHOLOGICAL** : rat model of chronic constriction injury, NeuN positive cells^94^ with antibody C. | **PATHOLOGICAL** : MS patients HLA-DR positive microglial cells^97^ with antibody L. MS patients, activated microglia-like cells^59^ with antibodies P and R. | **PATHOLOGICAL** : MS patients GFAP positive astrocytes^97^ with antibody L. | **PATHOLOGICAL** : MS patients CD45 and CD3-postive infiltrating macrophages and T cells^97^ with antibody L. |
|  | **HYPOTHALAMUS** | **PHYSIOLOGICAL** : NSE-positive neurons^41^ with antibodies A and u. | **PHYSIOLOGICAL** : Iba1-positive cells^38^ with antibody Q. **PATHOLOGICAL** : MDMA induced neuroinflammation in rat, CD11b-positive cells^92^ with antibody B. |  |  |

Regions where CB2R protein has been detected with antibodies that have not been fully validated in both physiological and pathological conditions are listed in the table. Abbreviations: AD, Alzheimer’s Disease; CA, cornu ammonis; CB2R, cannabinoid receptor type 2; CD, cluster differentiation; DG, Dentate Gyrus; mRNA, messenger ribonucleic acid ; GFAP, Glial fibrillary acidic protein; HD, Huntington’s Disease; HLA-DR, Human Leukocyte Antigen – DR isotype; Iba1, ionized calcium-binding adapter molecule 1; MDMA, 3,4-Methyl​enedioxy​methamphetamine; MS, Multiple Sclerosis ; NeuN, neuronal nuclei; NHP, non-human primate; NSE, Neuron Specific Enolase; PD, Parkinson’s Disease; TH, tyrosine hydroxylase; TLE, Temporal Lobe Epilepsy.

**Supplementary Table 3: Brain regions where CB2R protein, but not mRNA, was detected using unvalidated antibodies.**

| **REGIONS** | **NEURONS** | **ASTROCYTES** | **OTHER CELLS** |
| --- | --- | --- | --- |
| **COCHLEA** | Outer and inner hair cells^37^ with antibody C. |  |  |
| **CORPUS CALLOSUM** |  | GFAP-positive cells^63^ with antibody B. |  |
| **MIDBRAIN AND PONS NUCLEI** | Paratrochlear nucleus, paralemnniscal nucleus, red nucleus. Staining was also observed in the pontine nucleus, dorsal nucleus of lateral lemniscus, vestibular nucleus, dorsal cochlear nucleus, nucleus of spinal tract trigeminal nerve, oral part and lateral vestibular nucleus, parvocellular reticular nucleus and facial nucleus. No cellular marker was investigated to determine the identity of these CB2R-positive cells^92^ with antibodies A and u. Mice paratrochlear nucleus, paralemniscal nucleus, red nucleus, pontine nuclei, inferior colliculus, and the parvicellular portion of the medial vestibular nucleus^66^ with antibody v. | | |
| **LATERAL HABENULA** | Unidentified cells ^98^ with antibody M. | | |

Regions where CB2R protein has been detected with antibodies that have not been fully validated in physiological conditions in areas where the presence of CB2R mRNA has never been investigated to the best of our knowledge. Abbreviations: CB2R, cannabinoid receptor type 2; GFAP, Glial fibrillary acidic protein; mRNA, messenger ribonucleic acid.

**Supplementary Table 4. Summary of the brain areas likely to express CB2R protein**

|  | **mRNA+** | | **mRNA ?** | |
| --- | --- | --- | --- | --- |
|  | **Brain area** | **Cell types** | **Brain area** | **Cell types** |
| **Validated antibodies (D, G, H and K)** | Hippocampus, VTA, Red Nucleus, Brainstem, Retina | Neurons, microglia, astrocytes |  |  |
| **Unvalidated antibodies (A, B, C, E, F, I, J, L, M, N O, P, Q, R, s, t, u and v)** | Neocortex, Nucleus Accumbens, Striatum, Amygdala, Substantia Nigra, Cerebellum, Spinal Cord, Hypothalamus | Neurons, microglia, astrocytes | Lateral Habenula, Corpus callosum, Midbrain and Pons nuclei, Cochlea, | Astrocytes, ? |

The regions listed in the left columns (mRNA +) correspond to the regions for which studies have shown the presence of CB2R transcript (by RT-qPCR and/or in situ hybridization), as detailed in section 3. “**Brain regions and cell types found to express CB2R at the mRNA level**”. The regions listed in the right columns (mRNA?) correspond to the regions for which we have not identified any studies that have detected CB2R transcript. The letters assigned to the antibodies correspond to those assigned in Table 2. Abbreviations: CB2R, cannabinoid receptor type 2; mRNA, messenger ribonucleic acid; VTA, Ventral Tegmental Area.
